# Brittle Diabetes Following Partial Pancreatectomy: A Case of Type 3c Diabetes Mellitus Complicated by Type 4 Renal Tubular Acidosis

**DOI:** 10.7759/cureus.93267

**Published:** 2025-09-26

**Authors:** Yussif Abuharaz, Prattyak Mukhopadhyay, Faiz Saulat, Vijay Prabhakaran

**Affiliations:** 1 Internal Medicine, University of Florida College of Medicine, Gainesville, USA; 2 Internal Medicine, Veterans Affairs Medical Center, Gainesville, USA

**Keywords:** brittle diabetes, endocrinology and diabetes, renal tubular acidosis type 4, treatment of diabetes mellitus, type 3c diabetes mellitus

## Abstract

Type 3c diabetes mellitus (T3cDM), or pancreatogenic diabetes, is an under-recognized subtype of diabetes resulting from exocrine pancreatic disease or surgery. It is frequently misclassified as type 1 or type 2 diabetes, leading to inappropriate management. Brittle diabetes, characterized by severe glycemic variability and insulin sensitivity, is rare in T3cDM. Type 4 renal tubular acidosis (RTA), or hyperkalemic distal RTA, is another underdiagnosed complication of diabetic kidney disease. We report a unique case of transition to T3cDM presenting as brittle diabetes in a patient with a history of partial pancreatectomy, complicated by type 4 RTA.

This case highlights the clinical challenge of managing brittle diabetes in the context of T3cDM, a condition that often goes unrecognized in patients with a history of pancreatic surgery. The concurrent diagnosis of type 4 RTA complicated glucose and electrolyte management, as insulin is both therapeutic for hyperkalemia and a risk for hypoglycemia in brittle diabetes. Awareness of this overlap is essential, as both conditions require individualized therapy that departs from traditional inpatient diabetes management.

Clinicians should maintain a high index of suspicion for T3cDM in patients with pancreatic pathology and unstable glycemic profiles. Accurate diagnosis is key to appropriate treatment and prevention of complications. Additionally, type 4 RTA should be considered in diabetics with unexplained hyperkalemia despite preserved renal function. A multidisciplinary approach is essential for managing the complex interplay between glucose and potassium regulation in such patients.

## Introduction

This case highlights a patient with brittle diabetes and prior partial pancreatectomy who transitioned to type 3c diabetes mellitus (T3cDM), manifesting as markedly labile glycemia and exaggerated sensitivity to insulin. A case that underscores the importance of considering pancreatogenic diabetes in patients with unstable glycemic control and a history of pancreatic disease, trauma, or surgery [1]. 

Diabetes Mellitus is a heterogeneous disease with variable clinical presentations and complications. Type 1 and type 2 diabetes mellitus (T1DM, T2DM) represent most cases; however, other forms exist with increasing recognition, including T3cDM, or pancreatogenic diabetes [1,2]. T3cDM arises as a result of exocrine pancreatic disease, most commonly chronic pancreatitis, pancreatic surgery, neoplasm, or trauma [2,3]. It is often misclassified as T1DM or insulin-dependent T2DM, leading to challenges in management and recognition [2,3]. 

Brittle diabetes describes a subset of patients with highly unstable glycemic control characterized by severe swings in blood glucose levels and difficulty achieving stable control despite conventional therapy [4]. Brittle presentations are classically associated with autoimmune diabetes but may also occur due to secondary causes such as T3cDM [4].

Renal tubular acidosis (RTA) type 4, or hyperkalemic distal RTA, results from impaired aldosterone secretion or resistance, and is often associated with diabetic nephropathy [3,5]. The coexistence of brittle diabetes, T3cDM, and RTA type 4 has not been well documented in the literature. 

We present a case of a patient with a history of partial pancreatectomy and insulin-dependent T2DM, who demonstrated brittle diabetes with marked sensitivity to insulin, and was ultimately diagnosed with T3cDM. This case was further complicated by hyperkalemia due to newly diagnosed RTA type 4.

## Case presentation

A 64-year-old male with a history of insulin-dependent T2DM and prior partial pancreatectomy presented after outpatient labs revealed severe hyperkalemia. 

History 

The patient presented with a long history of insulin-dependent diabetes, labeled as T2DM, but with increasingly unstable glycemic control over the preceding year. He reported wide fluctuations in blood glucose despite adherence to his insulin regimen. He denied recent illness, vomiting, or diarrhea. Home medications included basal-bolus insulin, antihypertensives, and statin therapy. 

Surgical history was notable for a prior partial pancreatectomy due to pancreatic cysts. There was no history of autoimmune disease, chronic pancreatitis, or alcohol use disorder.

Presentation and initial evaluation 

On arrival at the emergency department (ED), the patient was hemodynamically stable. The physical exam revealed no acute distress, no signs of dehydration, and no Kussmaul respirations. 

Initial laboratory workup upon admission is summarized in Table 1. 

**Table 1:** Initial laboratory workup on admission. mg/dL, milligrams per deciliter; mmol/L, millimoles per liter; pH, potential of hydrogen (measure of acidity/alkalinity) Reference ranges adapted from the *American Diabetes Association Standards of Care in Diabetes-2025* [6]and *Tietz Textbook of Clinical Chemistry and Molecular Diagnostics* [7].

Lab	Result	Reference range	Units
Serum glucose	520	70-100	mg/dL
Serum potassium	6.2	3.5-5.0	mmol/L
Serum bicarbonate	18	22-28	mmol/L
Serum creatinine	1.2	0.6-1.3	mg/dL
Anion gap	Normal	8-12	mmol/L
Venous blood gas pH	7.29	7.31-7.41	-
Venous blood gas bicarbonate	18	22-28	mmol/L
Urinalysis: glucose (glycosuria)	Positive	Negative	-
Urinalysis: ketones (ketonuria)	Negative	Negative	-
Serum β-hydroxybutyrate	<0.10	<0.40	mmol/L

Given the absence of ketosis, normal anion gap, and absence of classic findings on physical exam, the patient did not meet diagnostic criteria for diabetic ketoacidosis (DKA) [4]. 

Hospital course 

The patient received 4 units of subcutaneous insulin aspart for hyperglycemia. Within hours, his blood glucose dropped to 46 mg/dL, necessitating immediate intravenous dextrose administration. This dramatic swing highlighted his insulin sensitivity and raised concern for brittle diabetes [4]. An insulin infusion was deferred to avoid recurrent severe hypoglycemia. 

Hyperkalemia was addressed with temporizing measures. The patient was given intravenous calcium gluconate, multiple rounds of potassium binder medications, insulin with glucose, and loop diuretics. Nephrology was consulted due to recurrent hyperkalemia in the setting of mild metabolic acidosis with relatively preserved renal function. 

Workup revealed findings consistent with type 4 RTA: persistent hyperkalemia despite preserved GFR, a non-anion gap metabolic acidosis, and normal plasma renin activity with low aldosterone concentration [3,5].

The etiology was presumed to be hyporeninemic hypoaldosteronism related to diabetic nephropathy.

Endocrinology was consulted for the management of diabetes. Review of the patient’s history of partial pancreatectomy, insulin dependence, and labile glucose suggested progression to type 3c diabetes, or pancreatogenic diabetes, rather than conventional T2DM. His brittle phenotype was attributed to loss of pancreatic β-cell reserve compounded by reduced glucagon-mediated counter-regulation following partial resection of the pancreas.

Follow-up and outcome 

The patient was treated with careful titration of subcutaneous insulin and potassium-lowering therapy. He was discharged on a tailored home sliding scale insulin regimen requiring close glucose monitoring. Outpatient follow-up with endocrinology confirmed the diagnosis of T3cDM, and nephrology initiated further management of type 4 RTA with potassium binders and diet modification. The patient’s potassium remained controlled, though his glucose variability persisted, requiring frequent insulin adjustments.

## Discussion

This case highlights several key teaching points. 

Recognition of T3cDM 

T3cDM accounts for 5%-10% of diabetes cases but is under-recognized, frequently misdiagnosed as T1DM or T2DM [1,2]. Diagnostic clues include a history of pancreatic surgery or trauma, low insulin requirements, unpredictable glucose swings, and concomitant exocrine pancreatic insufficiency [1,2]. Unlike T1DM, autoantibodies are absent, and unlike T2DM, there is often little insulin resistance [2]. In our patient, partial pancreatectomy and brittle glycemic control indicated a transition from insulin-dependent T2DM to T3cDM [1,2]. 

Brittle diabetes phenotype

Brittle diabetes is typically defined by extreme glycemic instability leading to frequent hospitalization [4]. In T3cDM, this arises from combined insulin deficiency and impaired glucagon response [2,4]. In our patient, dramatic hypoglycemia following only 4 units of aspart illustrates the exaggerated sensitivity [4]. 

Coexistence with type 4 RTA

Type 4 RTA is commonly seen in diabetics due to hyperreninemic hypoaldosteronism [3,5]. Patients may develop type 4 RTA before renal dysfunction reveals itself, and thus require a high index of suspicion in patients with a history of diabetes who now present with elevated serum potassium [8]. Hyperkalemia in this setting complicates therapy, as insulin is both a treatment for hyperkalemia and a precipitant of hypoglycemia [3,5]. Our case illustrates this paradox as insulin was needed to shift potassium but risked profound hypoglycemia [3,5]. 

Clinical guidelines and management implications 

Current guidelines from the American Diabetes Association (ADA) emphasize accurate diabetes classification, as management differs between subtypes [9]. Recognition of T3cDM is important to avoid potentially harmful treatment with insulin or oral agents [9]. Kidney Disease: Improving Global Outcomes (KDIGO) guidelines on hyperkalemia and metabolic acidosis in CKD highlight type 4 RTA as a common diabetic complication requiring chronic therapy with potassium binders, mineralocorticoid analogues, or dietary measures [10].

## Conclusions

This is a case of rare but important overlap of brittle diabetes secondary to a transition to T3cDM following a partial pancreatectomy, complicated by type 4 RTA. There should be a high index of suspicion among clinicians for T3cDM in patients with a history of pancreatic surgery and labile glycemia. Additionally, recognition of type 4 RTA as a cause of hyperkalemia in diabetics is essential, as this significantly impacts the use of insulin. Multidisciplinary care with endocrinology and nephrology was key to optimizing the management of this patient.
